# Supplementary material for: Maternity care providers’ experiences of work-related serious events (MATES): An International survey
Source: PLoS One. 2025 Feb 14;20(2):e0317682. doi: 10.1371/journal.pone.0317682 (PMC11828367; doi:10.1371/journal.pone.0317682)
Supplement: S2.File — (DOCX) [file pone.0317682.s002.docx]

# **S2 - Serious events**

Serious events mentioned by respondents on an open-ended question: *During your work as a maternity care provider, have you ever been directly involved in one of the following serious events? Exposure to any other severe event, such as …*

**Maternal obstetric emergencies, including:**

- Acute coronary syndrome
- Amniotic fluid embolism
- Covid 19
- Dangerous manipulation of a ventilation machine
- DIC
- Hysterectomy
- Maternal death (mentioned 4 times)
- Maternal emergencies (mentioned 5 times)
- Maternal Suicide (including attempted) (mentioned 4 times)
- Postnatal sepsis
- Perineal trauma
- PPH (mentioned 9 times)
- Psychosis/acute maternal mental health (mentioned 2 times)
- Traumatic birth experience (e.g. Instrumental Deliveries) (mentioned 2 times)

**Fetus related emergencies, including:**

- Diagnosed/Undiagnosed fetal abnormality (mentioned 2 times)
- Extreme prematurity (mentioned 2 times)
- Intrauterine death (mentioned 17 times)
- Late abortion with living baby
- Neonatal death (mentioned 2 times)
- Placenta abruption
- Resuscitation of the baby (mentioned 5 times)
- Shoulder dystocia (mentioned 4 times)
- Stillbirth (mentioned 9 times)
- Twin pregnancy with a vaginal birth in breech position

**Treatment of women, including:**

- Obstetric violence (mentioned 3 times)
- Discrimination (mentioned 2 times)

**Women’s Complex Social Circumstances, including:**

- Concealed pregnancy
- Death of a woman’s partner during pregnancy
- Domestic violence (including rape, incest) (mentioned 9 times)

**Maternity Care providers working experiences, including:**

- Aggression from obstetric staff towards midwifery staff (mentioned 3 times)
- Bullying
- Colleague suicide (mentioned 3 times)
- Insufficient number of staff (Short staff) (mentioned 2 times)
- Involved in Court case (mentioned 2 times)
- Managing staff involved in these events
- Staff trauma (mentioned 2 times)
- Witnessing maternal health professional’s verbal/physical abuse towards women (mentioned 5 times)
