# Supplementary material for: Maternity care providers’ experiences of work-related serious events (MATES): An International survey
Source: PLoS One. 2025 Feb 14;20(2):e0317682. doi: 10.1371/journal.pone.0317682 (PMC11828367; doi:10.1371/journal.pone.0317682)
Supplement: Attachment — Submitted filename: Response to Reviewers.docx [file pone.0317682.s003.docx]

**Response to Reviewers**

| Reviewer #1:  Comments | Author Comments | Manuscript changes –  Now reads: |
| --- | --- | --- |
| L 98 -100 The term 'positive experience' comes from the WHO (2018) which states that childbirth should be a positive life event to optimize the experience of childbirth for women and their babies while recognising that adverse events can and do occur. The context of this statement should be clarified which could be followed by the Beck reference. | Thank you for this comment, see amended sentences | *The World Health Organization (WHO) stresses that a positive childbirth experience should be the ultimate goal of maternity care for all women, even when medical interventions are necessary [1]. However, serious complications can occur throughout the perinatal period, with most happening during labour and birth [2].* |
| If this is an international study, perhaps the authors could present international data on adverse events (morbidity/mortality) – these are expectations for MCPs globally. | As this study focuses on maternity care providers’ experiences of work-related serious events and does highlight their risk of exposure (67%-90%) to a traumatic birth event. The authors suggest that relevant data is presented from the providers’ perspective. |  |
| L178. I presume that informed consent was by means of a check box to proceed with the online study, did the participants also submit a 'written consent'?  Also, explain what is meant by the term 'sufficient reflective time' for an online survey. | Thank you, we understand how these sentences require clarity.  No participants did not submit written consent, a check box was ticked to proceed with the online study. Participants could also return via the link to the online survey - See amended sentences. | *Potential participants entered the survey through an information page explaining the study, where they gave their approval via ticking a check box relating to an online statement of consent, indicating that they understood the information and agreed to participate in the study. This action was a requirement (set within Qualtrics^XM^) to enabled participants access to complete the survey, allowing participants sufficient reflection time.* |
| L 201. Were the 180 incomplete surveys included in the analysis? | Thank you for seeking clarification. Yes, the 180 incomplete surveys were included, as despite these participants not completing the whole survey they did answer key questions on being involved in serious events during maternity care. In addition, all findings presented within the tables are reported with the n= value clearly highlighted. |  |
| Provide more information on the 33 countries? Europe, Asia, Africa, Americas? Also a breakdown of the participants would be helpful, numbers of midwives, nurses, physicians, obstetricians, neonatologists, GPs, and mental health care providers. It seems most respondents nurse/midwives or physicians. | Thank you for this comment – further detail is provided in the manuscript. | *In total, 579 maternity care providers from 33 countries from Asia, Africia, Australia, Europe and North America participated in the study (See S1 for list of countries).*  *Respondents had various professional backgrounds including midwives (416), nurses (41), physicians (obstetricians (78), neonatologists (15), GPs (1)) plus other professionals (7) such as, mental health care providers (13) who cared for women during pregnancy, birth, and postnatal periods. Eight respondents did not mention their primary professional role.*  *Demographic characteristics of respondents (including primary professional role) are presented in S1.* |
| Consider the limitations - survey in English excluded potential participants, also bias in relation to publications in English (US, Australia, UK, Ireland, Nordic countries). | We agree with this comment – see additional insert on heading and sentence added. | ***Recommendations/Implications for practice/ Future Research/Limitations***  *A limitation of the study is that the survey was disseminated in English language to 33 countries via COST Action members and the results are also published in English.* |
| Reviewer #2:  Comments |  |  |
| Reviewer #2:   Table 7 is complex and worth explaining in words the key relationships and implications from the table. | Thank you, see additional explanation provided | *Therefore, experiencing aggression by the woman or her family increases the level of MCP burnout. More years of work experience and more confidence in providing care after the event are significantly associated with a decrease on the same ProQOL-subscale. Thereby the results suggests that more years of experience decreases the MCP’s level of burnout and their level of Secondary Traumatic Stress.* |
| In terms of future actions the authors may wish to inform themselves of the Trauma Risk Management (TRiM) TRIM programme which is being piloted in healthcare as a potential action to provide early support for colleagues.  D. Whybrow, N. Jones, N. Greenberg, Promoting organizational well-being: a comprehensive review of Trauma Risk Management, Occupational Medicine, Volume 65, Issue 4, June 2015, Pages 331–336, <https://doi.org/10.1093/occmed/kqv024> | Thank you for your suggestion – see amended sentence | *Programmes are being piloted [42] and following detailed evaluation of the impact, consideration regarding implementation is necessary to provide an effective peer support system for MCPs.* |
